# rhFGF-21 accelerates corneal epithelial wound healing through the attenuation of oxidative stress and inflammatory mediators in diabetic mice

**DOI:** 10.1016/j.jbc.2023.105127

**Published:** 2023-08-04

**Authors:** Le Li, Huan Wang, Shucai Pang, Liangshun Wang, Zhengkai Fan, Chunyu Ma, Shufen Yang, Joshua Banda, Qi Hui, Fangyi Lv, Haibing Fan, Tongzhou Huang, Xiaobi Zhang, Xiaojie Wang

**Affiliations:** 1 School of Pharmacological Sciences, Wenzhou Medical University, Chashan University Park, Wenzhou, China; 2 Department of Pharmacy, The Eye Hospital of Wenzhou Medical University, Wenzhou, China; 3 Laboratory of Zhejiang Province for Pharmaceutical Engineering and Development of Growth Factors, Collaborative Biomedical Innovation Center of Wenzhou, Wenzhou, China; 4 Research Units of Clinical Translation of Cell Growth Factors and Diseases, Chinese Academy of Medical Science, Wenzhou, China

**Keywords:** fibroblast growth factor 21, matrix metalloproteinase, cyclooxygenase, oxidative stress, superoxide dismutase, diabetic keratopathy, corneal epithelial wound healing, inflammatory response

## Abstract

Diabetic keratopathy, commonly associated with a hyperactive inflammatory response, is one of the most common eye complications of diabetes. The peptide hormone fibroblast growth factor-21 (FGF-21) has been demonstrated to have anti-inflammatory and antioxidant properties. However, whether administration of recombinant human (rh) FGF-21 can potentially regulate diabetic keratopathy is still unknown. Therefore, in this work, we investigated the role of rhFGF-21 in the modulation of corneal epithelial wound healing, the inflammation response, and oxidative stress using type 1 diabetic mice and high glucose–treated human corneal epithelial cells. Our experimental results indicated that the application of rhFGF-21 contributed to the enhancement of epithelial wound healing. This treatment also led to advancements in tear production and reduction in corneal edema. Moreover, there was a notable reduction in the levels of proinflammatory cytokines such as TNF-α, IL-6, IL-1β, MCP-1, IFN-γ, MMP-2, and MMP-9 in both diabetic mouse corneal epithelium and human corneal epithelial cells treated with high glucose. Furthermore, we found rhFGF-21 treatment inhibited reactive oxygen species production and increased levels of anti-inflammatory molecules IL-10 and SOD-1, which suggests that FGF-21 has a protective role in diabetic corneal epithelial healing by increasing the antioxidant capacity and reducing the release of inflammatory mediators and matrix metalloproteinases. Therefore, we propose that administration of FGF-21 may represent a potential treatment for diabetic keratopathy.

Diabetic keratopathy (DK) is one of the most common eye complications of diabetes. Clinically, DK manifests as a decrease in corneal sensitivity, a persistent epithelial defect, and delayed corneal epithelial wound healing (1, 2). The corneal epithelium acts as a barrier to protect the cornea and maintains ocular immune homeostasis by expressing anti-inflammatory molecules. Disruption of the epithelium has been shown to promote ocular inflammation in corneal disorders *via* the upregulation and abnormal secretion of proinflammatory cytokines interleukin (IL)-1β/6 and tumor necrosis factor-α (TNF-α) (3, 4). Chemokines play a crucial role in multiple inflammatory diseases (5). Several studies have demonstrated that the expression of interferon-γ (IFN-γ) and macrophage chemoattractant protein 1 (MCP-1) in inflamed corneal injury is escalated (6, 7). Following disruption, rapid re-epithelialization and restoration of the epithelial barrier are critical in maintaining the structural integrity and normal function of the cornea (8). In this process, elevated levels of reactive oxygen species (ROS) have been demonstrated to reduce epithelial barrier integrity and the expression of tight junction proteins (9). Furthermore, inflammatory factors such as TNF-α, ILs, and extracellular matrix metalloproteinases (MMPs) can lead to poor corneal functional and morphological changes (10, 11). Currently, the primary clinical treatment for DK with epithelial defects is based on preventing infection using prophylactic antibiotic eye drops and promoting epithelial healing using growth factor drugs like neurotrophic factor (12, 13, 14, 15). However, these drugs only provide temporary relief. Thus, there persists an unfulfilled demand for effective clinical solutions for the treatment of diabetic keratopathy (16, 17).

Fibroblast growth factor-21 (FGF-21) is a member of the fibroblast growth factor family. It is preferentially secreted by the liver and involved in both glucose and lipid metabolism (18). A study showed that FGF-21 can reduce blood sugar levels without causing hypoglycemic reactions and improves insulin sensitivity (19). Other studies found that FGF-21 can reduce the expression of inflammatory factors, including IL-6 and TNF-α, showing its potential anti-inflammatory effects (20, 21). Moreover, FGF-21 reduces lipopolysaccharide-induced ROS accumulation in macrophages and cardiomyocytes, thus suggesting it has antioxidant effects (22, 23). Our group previously demonstrated that FGF-21 enhances wound repair of scalded skin in diabetic rats by reducing the expression of inflammatory factors IL-6, IL-1β, and TNF-α while accelerating the transition of scalded skin from the inflammatory phase to the proliferative phase (24).

Based on the multiple functions of FGF-21 and the characteristics of diabetic corneal pathogenesis, we hypothesized that FGF-21 may be useful for DK treatment. To assess this hypothesis, we investigated the regulatory effects of recombinant human (rh) FGF-21 on corneal epithelial wound healing, the inflammatory response, and the antioxidant system, as well as the underlying mechanisms, using streptozotocin (STZ)-induced type 1 diabetic mice and high glucose–treated corneal epithelial cells.

## Results

### rhFGF-21 improves the viability and migration of human corneal epithelial cells under high glucose

**Figure 1:**
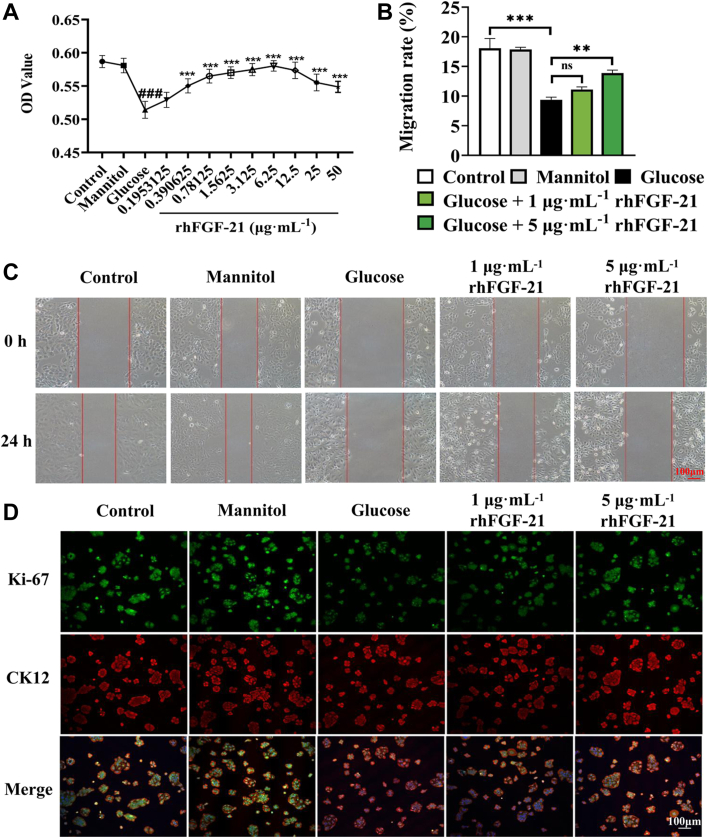
**Migration and proliferation of HCECs treated with glucose and rhFGF-21.***A*, cell proliferation after treatment with glucose in the absence or presence of rhFGF-21 for 24 h, as measured using the MTT assay. (∗represents statistic differences between glucose and glucose + rhFGF-21 groups, ∗*p* < 0.05, ∗∗*p* < 0.01, ∗∗∗*p* < 0.001; ^#^represents statistic differences between control and glucose, ^###^*p* < 0.001). *B*, migration rate was analyzed, and it is represented as the percentage of primary wounding area. *C*, confluent HCECs were wounded after treatment with 50 mM glucose. Cell migration was observed with or without rhFGF-21 treatment for 24 h. *D*, Ki-67 and CK12 expression, as determined by immunofluorescence analysis. (*n* = 6, ∗*p* < 0.05, ∗∗*p* < 0.01, ∗∗∗*p* < 0.001). CK12, Cytokeratin 12; FGF, fibroblast growth factor; HCEC, human corneal epithelial cell; MTT, methyl thiazolyl tetrazolium; rh, recombinant human.

To assess the effects of rhFGF-21 on human corneal epithelial cell (HCEC) wound healing, HCECs were cultured with 35 mM glucose with an equal concentration of mannose as the osmotic control and treated with or without rhFGF-21 for 24 h. Methyl thiazolyl tetrazolium (MTT) assay preliminary screening of glucose concentration revealed that 35 mM glucose decreased cell viability (Fig. S1). The MTT assay showed that rhFGF-21 protects against HCECs damage from high glucose (Fig. 1*A*). The viability of HCECs cultured in 35 mM glucose significantly increased following treatment with 0.39 μg mL^−1^ rhFGF-21 (*p* < 0.001), and the optimal rhFGF-21 concentration was about 6.25 μg mL^−1^ (*p* < 0.001) (Fig. 1*A*). Therefore, rhFGF-21 concentrations of 1 and 5 μg mL^−1^ were selected and used for subsequent experiments. Cell migration was assessed using the scratch assay. Treatment with 5 μg mL^−1^ rhFGF-21 promoted HCECs migration when compared with the glucose group (Fig. 1, *B* and *C*, *p* < 0.01). Ki-67 are well-known markers of re-epithelialization of injured corneas. We then examined the biological characteristics of the corneal epithelial cells using double immunofluorescence staining for cornea-specific Cytokeratin 12 and Ki-67. We observed that rhFGF-21 upregulates Ki-67 thereby suggesting it promotes re-epithelialization (Fig. 1*D*).

### rhFGF-21 significantly decreases the expression of proinflammatory cytokines and MMPs in HCECs under high glucose

**Figure 2:**
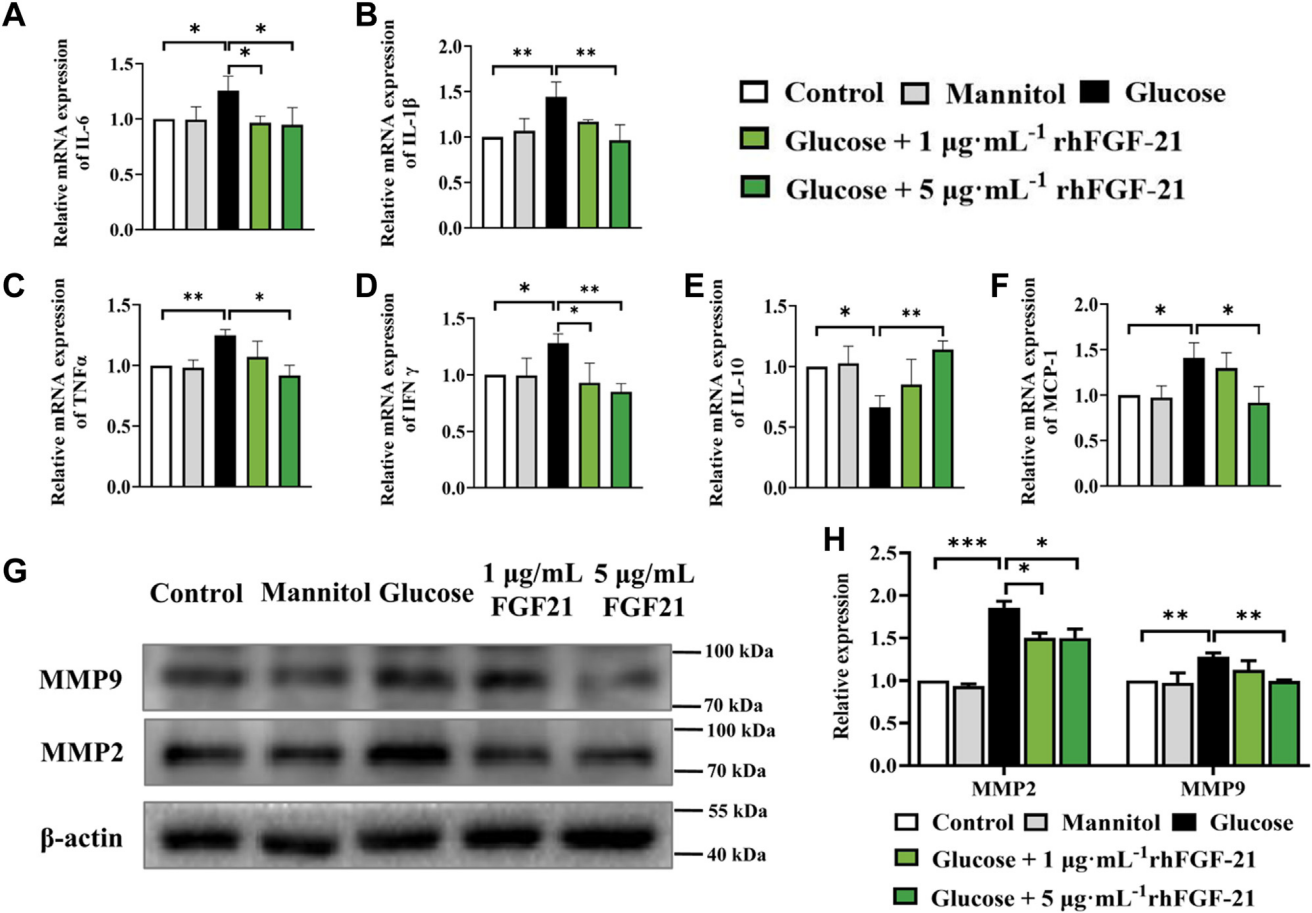
**Proinflammatory cytokines and MMPs concentrations in HCECs in the control, mannitol, glucose, and glucose + rhFGF-21 groups.***A*–*F*, cytokine profiles (TNF-α, MCP-1, IFN-γ, and IL-1β/6/10) of HCECs by qPCR. Numbers show relative mRNA expression. *G*, Western blotting of MMP-2 and MMP-9 expression of total protein extracts of HCECs. *H*, Western blot analysis.(*n* = 6, ∗*p* < 0.05, ∗∗*p* < 0.01, ∗∗∗*p* < 0.001). FGF, fibroblast growth factor; HCEC, human corneal epithelial cell; IL, interleukin; IFN, interferon; MCP, macrophage chemoattractant protein; MMP, matrix metalloproteinase; qPCR, quantitative real-time PCR; rh, recombinant human; TNF, tumor necrosis factor.

Since excessive inflammation is known to abrogate corneal re-epithelialization, we investigated whether rhFGF-21 affects the expression levels of inflammation-related factors IL-6, IL-1β, TNF-α, IFN-γ, MCP-1, IL-10, and MMPs (MMP-2 and MMP-9) in HCEC. Analysis of mRNA expression using quantitative real-time PCR (qPCR) in the control, mannitol, glucose, and glucose + rhFGF-21 groups showed that glucose treatment enhanced the expression of inflammatory factors IL-6, IL-1β, TNF-α, IFN-γ, and MCP-1. Treatment with rhFGF-21 attenuated the expression of these inflammatory factors restoring it to levels similar to those in the control group (Fig. 2, *A*–*D*, and *F*). Furthermore, rhFGF-21 administration increased the expression of IL-10, an anti-inflammatory cytokine, and regulator of oxidation, which was reduced after glucose treatment (Fig. 2*E*). Western blot analysis revealed that rhFGF-21 also significantly reduced glucose-induced MMP-2 and MMP-9 upregulation in a concentration-dependent manner (Fig. 2, *G* and *H*).

### rhFGF-21 attenuates hyperglycemia-induced oxidative stress in HCECs

**Figure 3:**
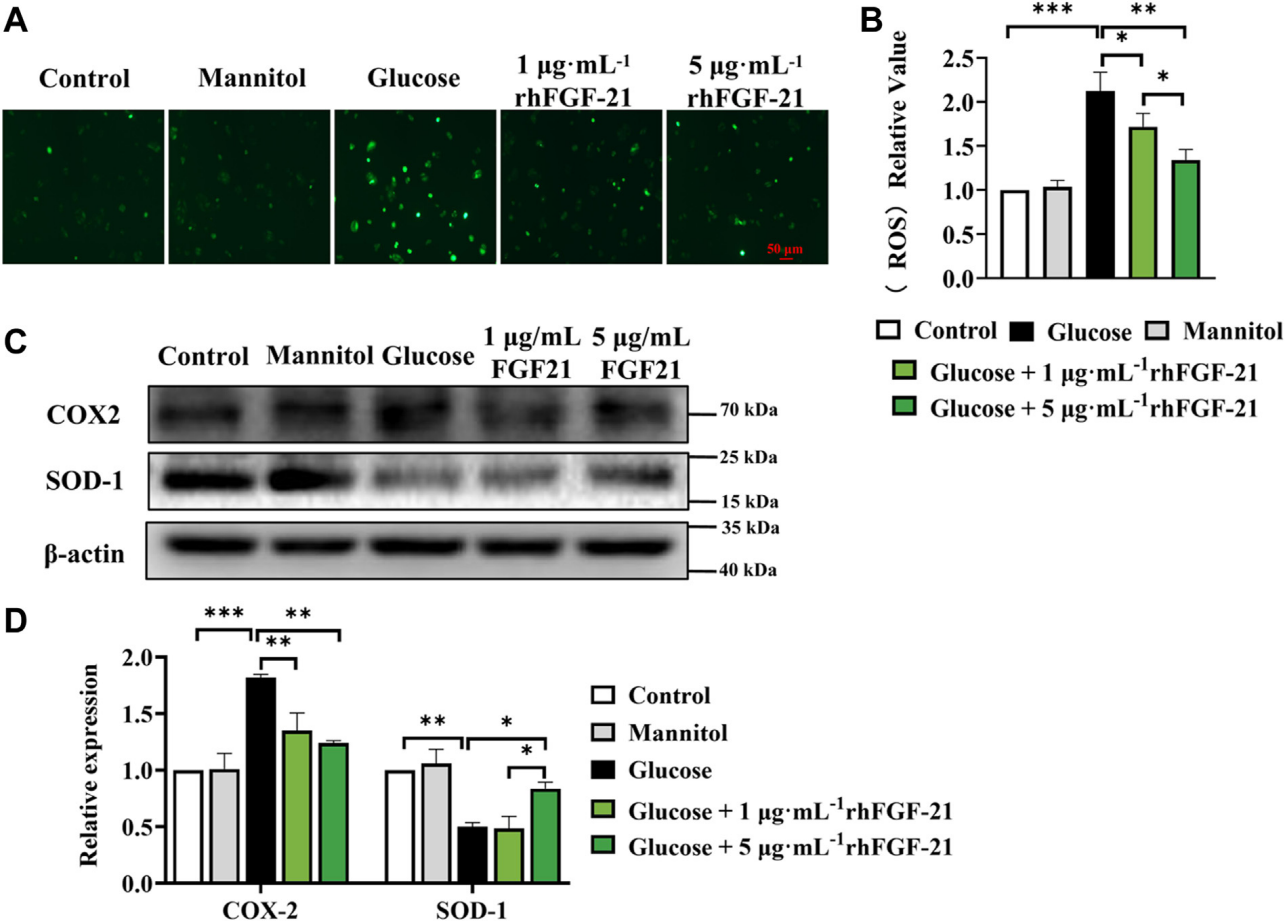
**ROS production, SOD-1, and COX-2 levels in hyperglycemia-induced HCECs in the control, mannitol, glucose, and rhFGF-21 groups.***A*, ROS production in HCECs. *B*, the relative value of ROS. *C*, Western blotting of SOD-1 and COX-2. *D*, expression levels of SOD-1 and COX-2 (*n* = 6, ∗*p* < 0.05, ∗∗*p* < 0.01, ∗∗∗*p* < 0.001). COX, cyclooxygenase; FGF, fibroblast growth factor; HCEC, human corneal epithelial cell; rh, recombinant human; ROS, reactive oxygen species; SOD, superoxide dismutase.

To evaluate the regulatory effects of rhFGF-21 on hyperglycemia-induced oxidative stress in HCECs, we measured cellular ROS production using the Reactive Oxygen Species Assay Kit and observed that rhFGF-21 attenuated ROS levels in a dose-dependent manner (Fig. 3, *A* and *B*). To further confirm the effects of rhFGF-21 on oxidative stress, we analyzed the expression of superoxide dismutase-1 (SOD-1) and cyclooxygenase-2 (COX-2) in HCECs using Western blot. SOD-1 and COX-2 are key biomarkers of redox balance. SOD-1 protects cells from oxidative damage, while COX-2 arbitrates oxidative stress. There were significantly higher SOD-1 levels and lower COX-2 levels detected in HCECs after rhFGF-21 treatment (Fig. 3, *C* and *D*). These results suggest that rhFGF-21 attenuates hyperglycemia-induced oxidative stress in HCECs.

### rhFGF-21 accelerates corneal wound healing and tear production in type 1 diabetic mice

**Figure 4:**
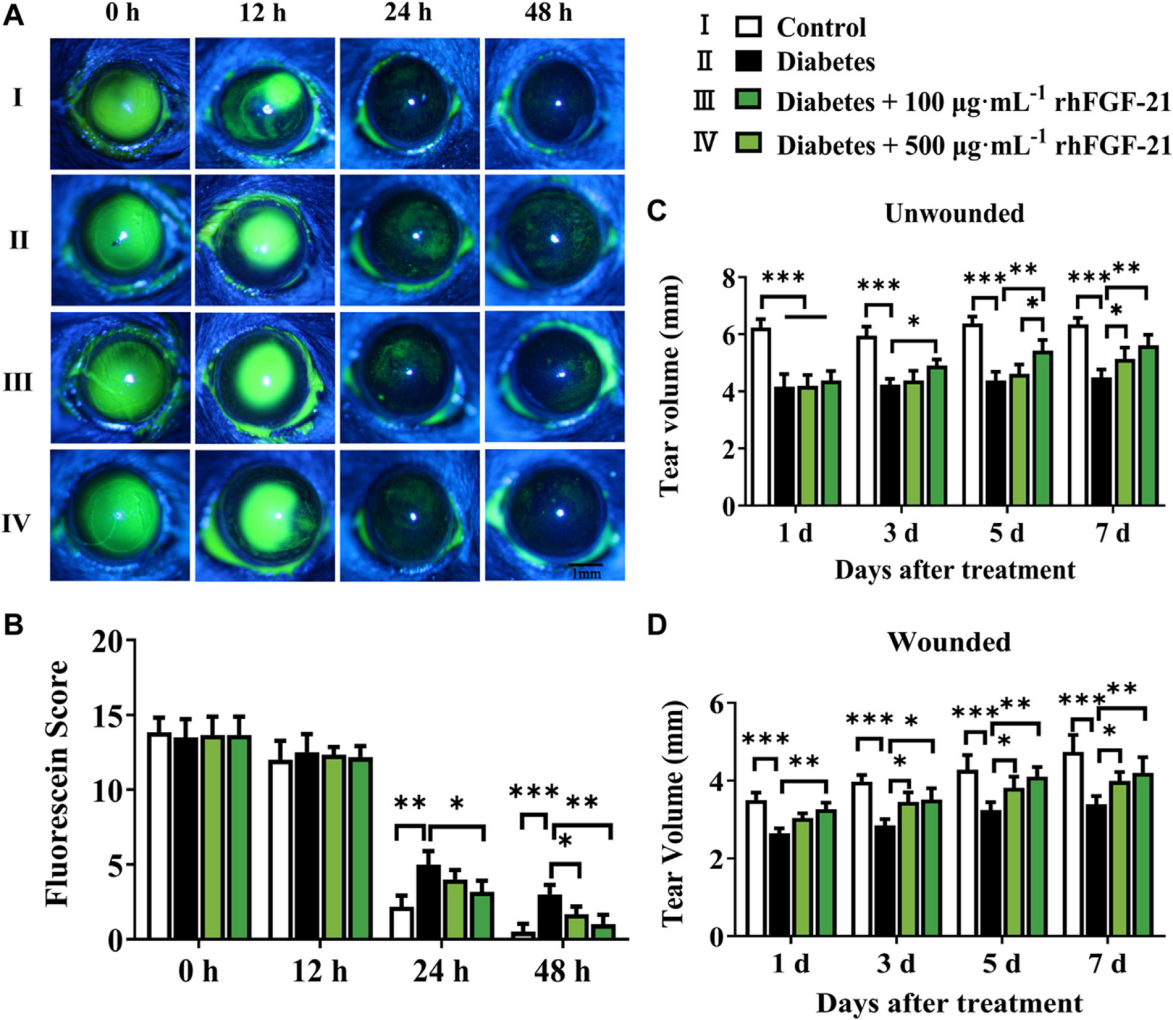
**Effects of topical application of rhFGF-21 on corneal wound healing and tear production in diabetic mice.***A*, corneal epithelial wound was stained with fluorescein sodium at 12, 24, and 48 h after debridement. *B*, residual epithelial defect is presented as the percentage of the original wound area. *C* and *D*, tear volume in unwounded eyes (*C*) and wounded eyes (*D*) of diabetic mice treated with rhFGF-21 for different periods (*n* = 6, ∗*p* < 0.05, ∗∗*p* < 0.01, ∗∗∗*p* < 0.001). FGF, fibroblast growth factor; rh, recombinant human.

A type 1 diabetic mouse model was generated by a single intraperitoneal injection of STZ. Blood glucose levels and body weight were measured at the indicated time points. The blood glucose levels in diabetic mice were significantly higher than those in control mice after 2 weeks of STZ injection (Fig. S2*A*) while the body weight decreased (Fig. S2*B*). Tear production was analyzed at 10 weeks; compared with the control group, tear production was significantly reduced in the diabetes group (Fig. S2*C*). We investigated the effects of rhFGF-21 on epithelial wound closure in the cornea, and our findings show that 500 μg mL^−1^ rhFGF-21 significantly accelerates corneal epithelial wound healing in diabetic mice after 24 h treatment (Fig. 4*A*). The wounds were healed in the 500 μg mL^−1^ rhFGF-21 group 48 h post-treatment (Fig. 4*B*). However, there we observed significant unhealed areas in the diabetes group. We then examined the effects of rhFGF-21 on tear production in diabetic mice. The right cornea of diabetic mice was injured, whereas the left eye was left unwounded. Compared with vehicle treatment, rhFGF-21 treatment groups significantly increased tear production in both the unwounded eyes (Fig. 4*C*) and wounded eyes (Fig. 4*D*) in diabetic mice.

### rhFGF-21 ameliorates corneal edema in diabetic mice

**Figure 5:**
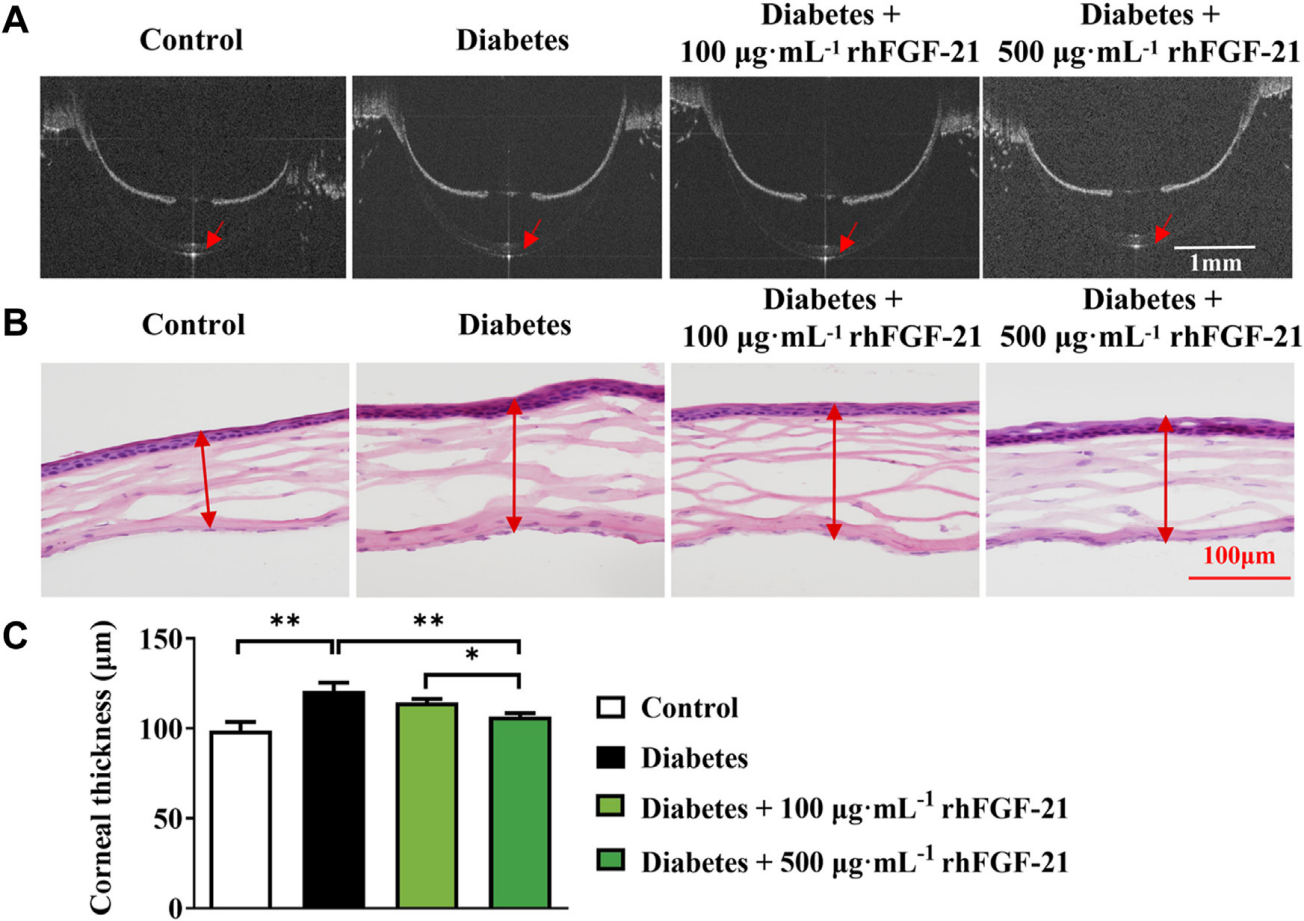
**Anterior segment OCT and immunohistochemical results.***A*, central corneal thickness, as measured by anterior segment OCT (7 days post-wounding). *B*, representative H&E staining of mouse corneal sections. *C*, statistical analysis of corneal thickness from panel A (*n* = 6, ∗*p* < 0.05, ∗∗*p* < 0.01). OCT, optical coherence tomography.

Seven days after corneal epithelial curettage, optical coherence tomography scanning of the anterior segment was performed in each group, and the central corneal thickness was quantified using the Image J software (https://imagej.net/ij/download.html). The cornea of diabetic mice was significantly thicker than that of normal mice (Fig. 5, *A* and *C*). Continuous administration of rhFGF-21 for 7 days attenuated corneal edema. These effects were more pronounced in the higher dose (500 μg mL^−1^ rhFGF-21) than in the lower dose (100 μg mL^−1^ rhFGF-21). H&E staining revealed that the cornea was thicker and the cell gap was increased in the diabetic group, while treatment with rhFGF-21 decreased the thickness of the cornea, restoring thickness to normal levels (Fig. 5*B*).

### rhFGF-21 improves corneal epithelial viability in diabetic mice

**Figure 6:**
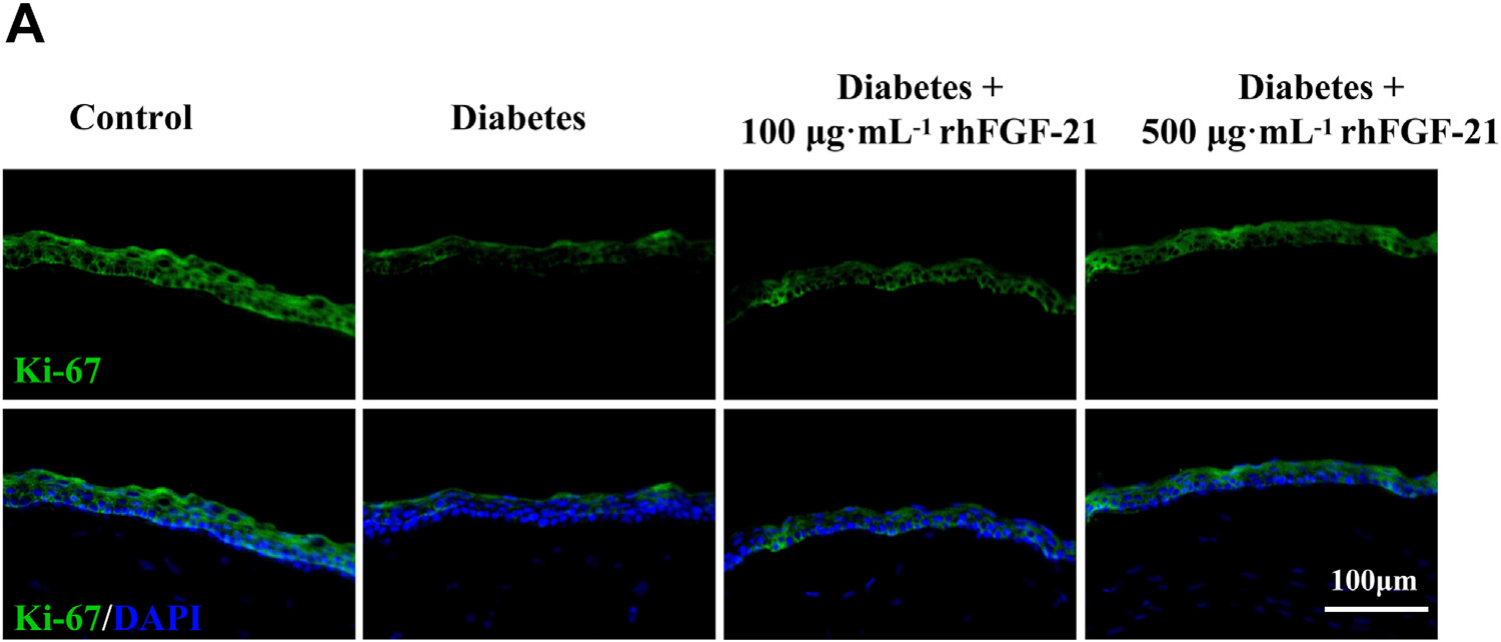
**Ki-67 immunofluorescence.***A*, Ki-67 immunofluorescence staining was performed at 24 h after corneal epithelial injury. (*n* = 6).

To evaluate the effects of rhFGF-21 on corneal epithelial proliferation in diabetic mice, corneal staining for Ki-67 was performed 24 h after injury. As shown in Figure 6, the Ki-67 fluorescence intensity was significantly decreased in the diabetic group compared with the control group, and the administration of rhFGF-21 increased the expression of Ki-67 in a dose-dependent manner. Treatment with 500 μg mL^−1^ rhFGF-21 was more effective than treatment with 100 μg mL^−1^ rhFGF-21. These results indicate that 500 μg mL^−1^ rhFGF-21 could significantly improve hyperglycemia-induced corneal epithelial damage.

### rhFGF-21 reduces the expression of inflammatory factors and MMPs in diabetic mice

**Figure 7:**
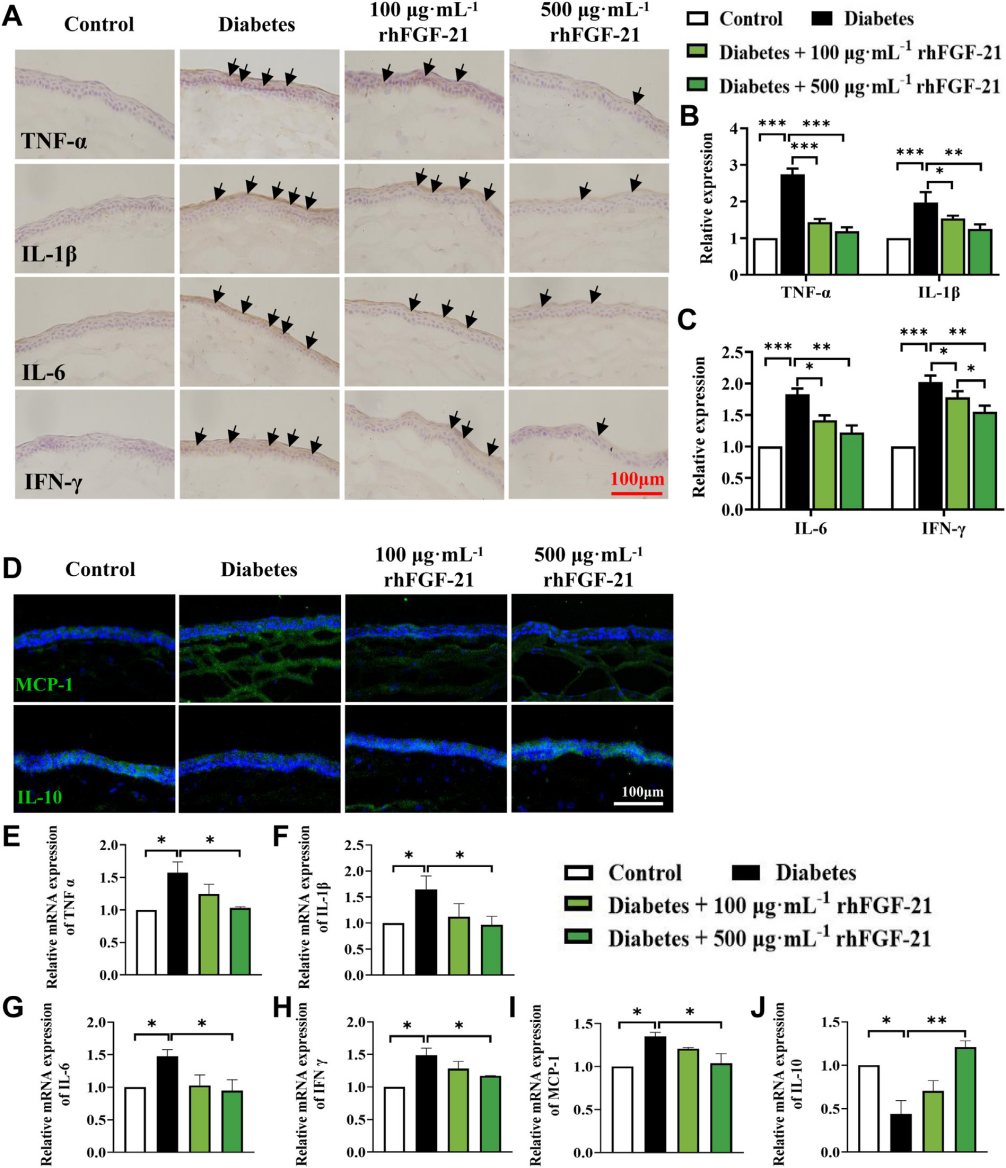
**Expression of inflammatory factors in diabetic mice with or without rhFGF-21 treatment.** Cytokine profiles (TNF-α, MCP-1, IFN-γ, and IL-1β/6/10) expression was determined by immunohistochemistry (*A*–*C*), immunofluorescence staining (*D*) and qPCR, numbers show relative mRNA expression (*E*–*J*). (*n* = 4 corneas mixed) (*n* = 6, ∗*p* < 0.05, ∗∗*p* < 0.01, ∗∗∗*p* < 0.001). FGF, fibroblast growth factor; IL, interleukin; IFN, interferon; MCP, macrophage chemoattractant protein; qPCR, quantitative real-time PCR; rh, recombinant human; TNF, tumor necrosis factor.

**Figure 8:**
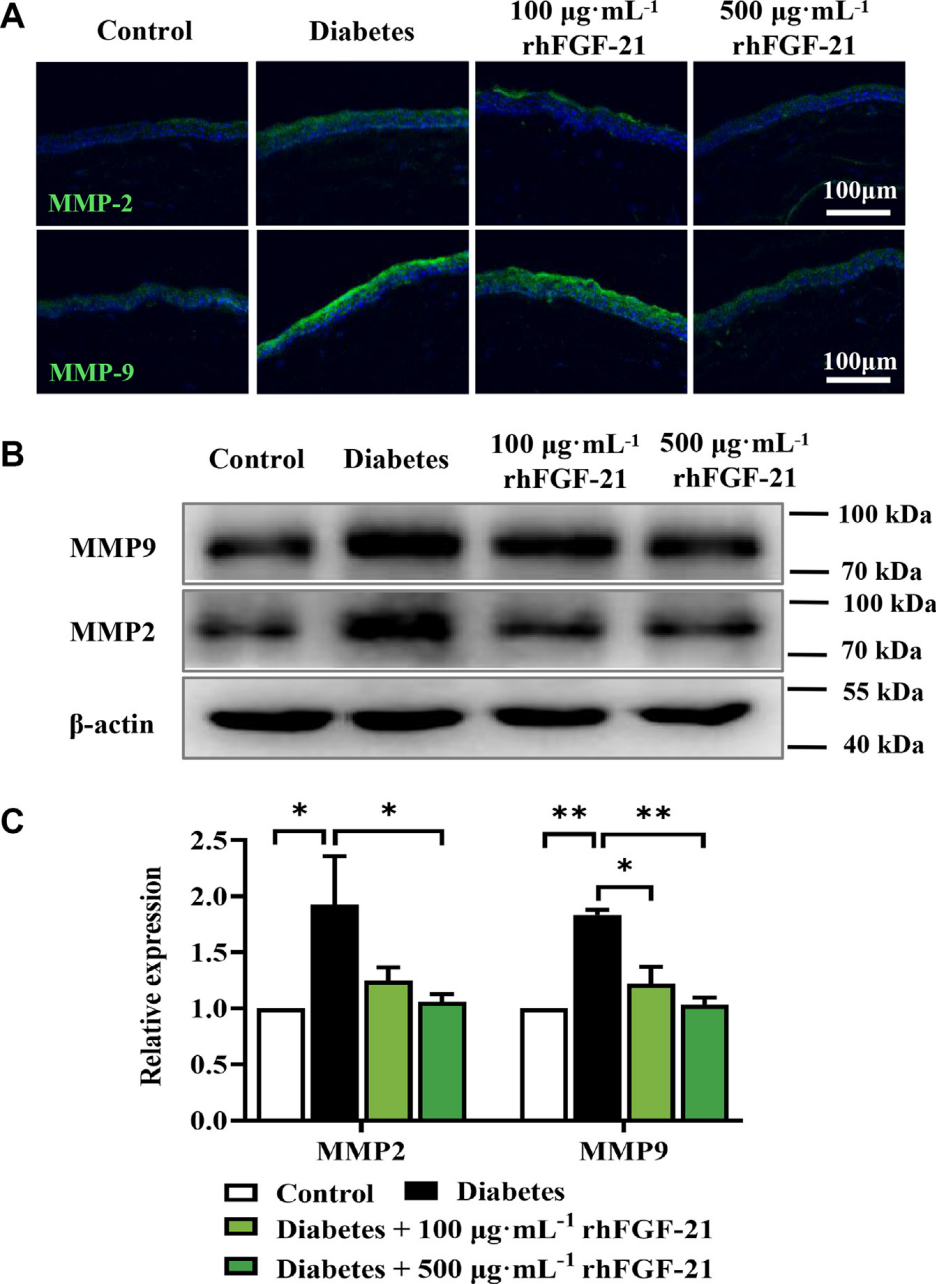
**Expression of MMPs in diabetic mice with or without rhFGF-21 treatment.***A*, immunofluorescence staining of MMP-2 and MMP-9. *B*, Western blotting of MMP-2 and MMP-9. *C*, Western blot analysis of MMP-2 and MMP-9. (*n* = 6, ∗*p* < 0.05, ∗∗*p* < 0.01). FGF, fibroblast growth factor; MMP, matrix metalloproteinase; rh, recombinant human.

To investigate whether rhFGF-21 accelerates corneal epithelial wound closure and tear secretion, we conducted a comprehensive investigation. This involved assessing its impact on the expression of MMPs, proinflammatory markers, cytokines, and chemokines. We employed a combination of techniques including immunohistochemistry, immunofluorescence, quantitative polymerase chain reaction (qPCR), and Western blotting in a diabetic mouse model. Immunohistochemistry and qPCR analysis of TNF-α, IL-1β, IL-6, and IFN-γ expression in the cornea revealed that rhFGF-21 treatment abolished inflammation by reducing the expression of these factors (Fig. 7, *A*–*C* and *E*–*H*). Accordingly, immunofluorescence and qPCR revealed that MCP-1 expression was markedly reduced, while IL-10 expression was elevated following rhFGF-21 treatment (Fig. 7, *D*, *I*, and *J*). Analysis of MMP-2 and MMP-9 using immunofluorescence and Western blot showed observations consistent with the *in vitro* results; rhFGF-21 significantly reduced MMP-2 and MMP-9 expression levels *in vivo* (Fig. 8, *A*–*C*). These results demonstrate that rhFGF-21 mitigates the expression of inflammatory factors, cytokines, chemokines, and MMPs and enhances IL-10 expression in diabetic mice.

### rhFGF-21 attenuates hyperglycemia-induced oxidative stress of the corneal epithelium in diabetes mice

**Figure 9:**
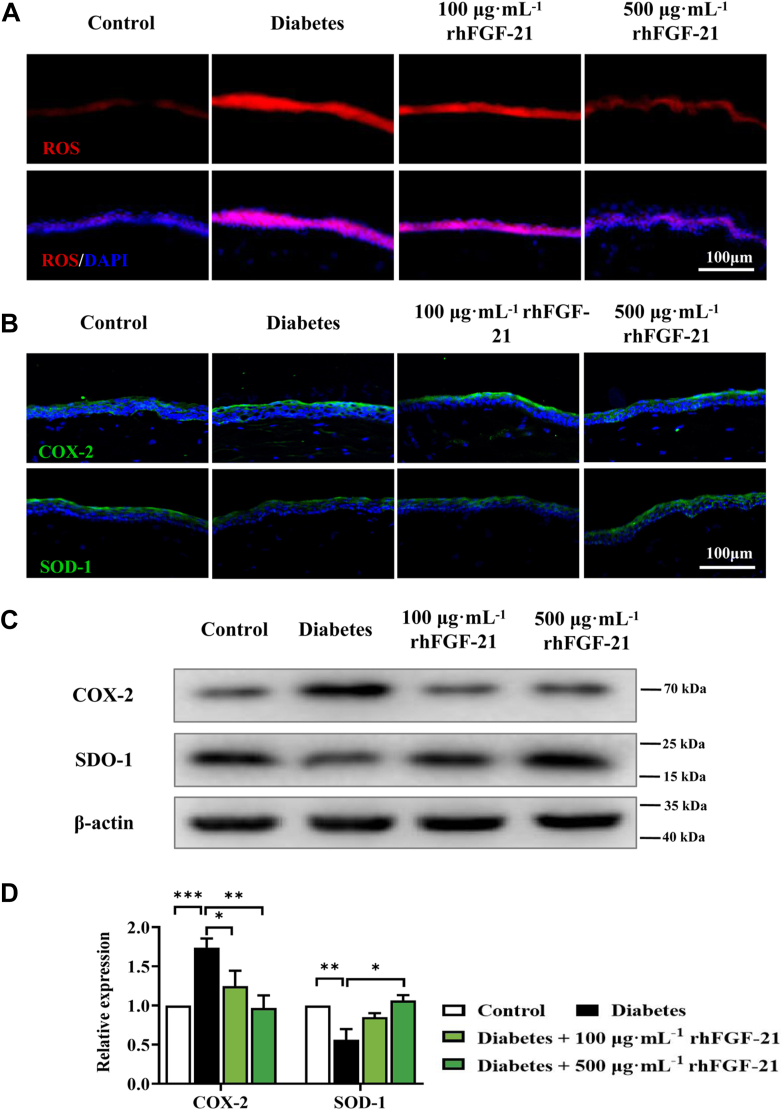
**ROS production and expression of COX-2 and SOD-1 in diabetic mice with or without rhFGF-21 treatment.***A*, ROS level in corneal epithelial tissue. COX-2 and SOD-1 expression was determined by immunofluorescence staining (*B*) and Western blot (*C* and *D*) (*n* = 6, ∗*p* < 0.05, ∗∗*p* < 0.01, ∗∗∗*p* < 0.001). COX, cyclooxygenase; FGF, fibroblast growth factor; rh, recombinant human; ROS, reactive oxygen species; SOD, superoxide dismutase.

To test the effects of rhFGF-21 on the oxidative stress response after corneal epithelial injury in diabetic mice, we measured ROS production in corneal epithelial using dihydroethidium (DHE) staining and the expression of SOD-1 and COX-2 by Western blot and immunofluorescence. The expression levels of ROS was markedly reduced in the 100 and 500 μg mL^−1^ rhFGF-21 groups compared with the diabetes group (Fig. 9*A*). Furthermore, the expression level of COX-2 was greatly increased in the diabetes group, whereas rhFGF-21 administration decreased the expression of COX-2. We also found that rhFGF-21 restored SOD-1 expression levels (Fig. 9, *B*–*D*). Altogether, our results indicate that rhFGF-21 has protective effects on oxidative stress in diabetic mice.

## Discussion

DK is a common ocular complication of diabetes and is reported in more than 70% of diabetic patients. Diabetes patients often suffer from persistent epithelial defects, corneal edema, and insufficient tear secretion (25, 26, 27), all of which contribute to DK. Therefore, the identification of therapeutics that alleviate these conditions is key to the development of novel DK treatment therapies. The present findings suggest that rhFGF-21 is a potent therapeutic for DK. We show that rhFGF-21 accelerates corneal epithelial wound healing, improves corneal edema and tear production, decreases the expression levels of TNF-α, IL-6, IL-1β, IFN-γ, MCP-1, MMP-2, and MMP-9, and increases IL-10 and SOD-1 expression in both the diabetic mice corneal epithelium and high glucose–treated HCECs.

FGF-21 plays a key role in mediating a variety of biological activities. However, its role in corneal epithelial wound healing remains unknown. Migration and proliferation of epidermal cells are essential for epidermal regeneration during wound healing. In this study, we found that treatment with rhFGF-21 significantly promoted HCECs migration and increased the expression of Ki-67 when compared with the glucose group.

Abnormal corneal epithelial barrier function is the pathological basis for several ocular diseases. It has been reported that during prolonged hyperglycemia, glucose causes auto-oxidation of proteins and induces the production of ROS (28). High glucose levels decrease the antioxidant defense and enhance oxidative stress in cells (29). After a corneal injury, in diabetic mice, inflammation and oxidative stress greatly limit the healing of the corneal epithelium and increase the risk of persistent defects of the corneal epithelium (1, 2). In addition, the corneal epithelium exhibits high levels of MMPs, which change the extracellular matrix environment and affect tissue repair during wound closure. Specifically, MMP-2 and MMP-9 levels are increased in tears and on the ocular surface during corneal wound closure (30, 31). We found that proinflammatory factors, chemokines and cytokines (TNF-α, IL-1β, IL-6, IFN-γ, and MCP-1), and MMPs (MMP-2 and MMP-9) were downregulated; anti-inflammatory properties of IL-10 was increased following rhFGF-21 treatment in high glucose–induced HCECs and diabetic mice with corneal wounds. Additionally, rhFGF-21 attenuated oxidative stress by restoring SOD-1 and downregulating COX-2. Taken together, these results suggest that FGF-21 may promote corneal epithelial repair by reducing the levels of inflammatory mediators and activating the antioxidant system. Furthermore, by reducing inflammation and oxidative stress, FGF-21 potentially resolves abnormal corneal epithelial function.

Corneal nerves are key components of the physiological system that controls ocular surface homeostasis. N. Morishige *et al* reported that diabetes reduces tear secretion and corneal sensation by damaging corneal nerve fibers (32). The amount of tear secretion in diabetic mice with corneal epithelial wounds was lower than that in normal mice. This may be due to corneal nerve damage further aggravating the decrease in tear production induced by high glucose levels. Yan *et al*. found that FGF-21 can induce sympathetic activity and protect nerves (33). Our results also confirmed the role of FGF-21 in repairing ocular surface nerves on the scopolamine-induced dry eye model (data to be published in another article). In the present study, we found that rhFGF-21 increased tear production in wounded and unwounded diabetic mice which may be due to the acceleration of corneal nerve regeneration and the reduction of high glucose–induced corneal damage.

In conclusion, we successfully established a diabetes mellitus type I mouse model exhibiting weight loss, elevated blood glucose levels, and reduced tear secretion. We elucidate the effects of rhFGF-21 on cell migration and viability using an *in vitro* model of hyperglycemia-induced corneal cells. Our findings show that rhFGF-21 effectively (i) alleviates inflammation by reducing the expression of inflammatory factors, cytokines and chemokines like IL-6, IL-1β, TNF-α, IFN-γ, and MCP-1, and increasing the expression of anti-inflammatory factors like IL-10 and (ii) attenuates the accumulation of ROS, by decreasing COX-2 and increasing SOD-1 expression, in both diabetic mice with corneal wounds and high glucose–induced HCECs. Furthermore, we show that rhFGF-21 accelerates corneal epithelial wound healing and improves corneal edema and tear production. These results may serve as a foundation for the potential use of rhFGF-21 in the clinical treatment of DK.

## Experimental procedures

### Animals

Male C57BL/6 mice (6–8 weeks old) weighing 19.4 to 22.8 g were purchased from Shanghai SLAC Laboratory Animal Co., Ltd. The animals were adaptively fed with free drinking water at the Experimental Animal Center of Wenzhou Medical University. All animals were approved by the Animal Institutional Ethics Committee of Wenzhou Medical University (approval ID wydw2022-0659). Type 1 diabetes mice were developed by intraperitoneal injection of STZ (60 mg kg^−1^) in a 10 mg mL^−1^ sodium citrate buffer solution (pH 4.2–4.5, enzyme grade; Thermo Fisher Scientific) for 5 days. The weight and blood glucose levels were recorded on the sixth day. The blood glucose levels were measured using a digital blood glucometer (Sinocare Inc) and mice with high blood glucose levels (>350 mg dL^−1^) were used. The weight and blood glucose levels were measured at 2, 4, 6, 8, and 10 weeks. Animals were randomly distributed into the following groups (*n* = 12 per group): the control group, the diabetes group, the diabetes + 100 μg mL^−1^ rhFGF-21 group, and the diabetes + 500 μg mL^−1^ rhFGF-21 group. rhFGF-21 was obtained from the Key Laboratory of Biotechnology Pharmaceutical Engineering of Zhejiang Province. Mice in the control and the diabetes group were intraperitoneally injected with saline of the same volume as rhFGF-21.

### Cell culture

HCECs (Bena Culture Collection Biotechnology Co, Ltd) were cultured in EMEM (Eagle's Minimal Essential Medium, Gibco) supplemented with 10% fetal bovine serum (Lonsera) and 100 U mL^−1^ penicillin (Phygene). At 60% confluence, the cells were starved for 24 h in a serum-free medium, followed by stimulation with 35 mM glucose or mannitol (Sigma-Aldrich) (35 mM mannitol as osmotic control) in the same serum-free medium for 24 h, with or without rhFGF-21.

### Cell viability assay

HCECs were starved overnight in bovine pituitary extract–free keratinocyte serum-free medium, treated with high glucose or mannitol for 24 h in the absence or presence of rhFGF-21 (1 μg mL^−1^ or 5 μg mL^−1^), and incubated with 20 μl MTT (Solarbio) for 2 h. The optical density value was measured at 450 nm using a microplate reader (Molecular Devices, LLC).

### Scratch wound healing assay

In the scratch wound healing assay, 3 × 10^5^ cells per well were plated in triplicate in a six-well culture plate and allowed to adhere overnight. At 80% confluence, the cells were starved for 24 h in a serum-free medium and then stimulated with 35 mM glucose for 24 h with or without rhFGF-21. A scratch was made using a 200-μL pipette tip to create a cell-free region across each well. After the scratch, the cultures were gently washed using PBS to remove detached cells. The cells were imaged at 0 and 24 h, and the images were processed using GraphPad Prism 6 Software (GraphPad Software, https://www.graphpad.com/dl/96314/10B92408/). Relative wound closure was quantified by determining the area between the cell borders within a defined region. Measurements were normalized to the area between cell borders at 0 h (immediately following the scratch).

### Measurement of tear volume

Tear volume was measured using a phenol red-soaked cotton thread (Tianjin Jingming New Technology Development Co, Ltd) that was applied using forceps in the lateral canthus for 15 s. The wetting length of the thread (shows tear volume) was read in a blinded approach under a microscope using a ruler offered by the manufacturer. Tear volume was measured on days 1, 3, 5, and 7 after corneal wounding.

### Corneal epithelial wound healing

All mice were anesthetized by an intraperitoneal injection of 1.0% (w/v) pentobarbital plus proparacaine. Then, a 2-mm circular wound was created using a trephine in the central cornea, and the epithelium was removed using an Algerbrush II Epithelial scraper (Alger). After surgery, the cornea of injured eyes were treated topically with Levofloxacin Eye Drops (ShenTian Pharmaceutical Co, Ltd) to prevent infection.

### Corneal fluorescein sodium staining

The central corneal epithelial defect area was measured at 0, 12, 24, and 48 h with topical anesthesia after injury. Staining was performed by dropping 0.5% fluorescein sodium, and images were taken with a digital camera. The cornea was divided into four quadrants for grading the fluorescein staining. Each quadrant was given a staining score as follows (34): positive fluorescein plaque, 4; very dense dot-like fluorescence, 3; dense dot-like pattern, 2; slightly fluorescein-resembling sparse dots, 1; and no fluorescence, 0. The scores were added up and examined for each eye.

### Immunofluorescence staining

For *in vivo* experiments, mouse eyes were enucleated and dehydrated with 10% and 20% sucrose solution at 4 °C for 4 h and overnight, respectively, embedded in Tissue-Tek optimal cutting temperature compound (Tissue-Tek, Sakura Finetek), and stored at −80 °C. Fresh frozen mouse eye tissues were sectioned to 5 μm and blocked with 5% bovine serum albumin (BSA) for 1 h at 37 °C and incubated with primary antibody at 4 °C overnight, followed by incubation with secondary antibody at 37 °C for 1 h. For *in vitro* experiments, HCECs were first fixed with 4% paraformaldehyde at 4 °C for 10 min and then permeabilized with 0.2% Triton X-100 (Solarbio) at room temperature for 10 min. The cells were then blocked with 5% BSA for 1 h at 37 °C and incubated with primary antibody at 4 °C overnight, followed by incubation with secondary antibody at 37 °C for 1 h. The following primary antibodies were used: anti-Cytokeratin 12 (Santa Cruz Biotechnology), anti-Ki-67 (Affinity Biosciences), anti-IL-10, anti-MCP-1, anti-COX-2, anti-SOD-1, anti-MMP-2, and anti-MMP-9 (Proteintech Group). The secondary antibody was CoraLite488-conjugated Goat Anti-Rabbit IgG(H+L) or Fluorescein (FITC)-conjugated AffiniPure Goat Anti-Rabbit IgG(H+L) (Proteintech Group). Samples were observed using a Nikon fluorescence microscope (ECLIPSE Ni-U) after counterstaining with DAPI (fluorescent DNA-binding dye). The mean fluorescent intensity of each image was analyzed using Image Pro Plus V.6.0.

### Histopathology and immunohistochemistry of mouse corneas

Mouse eye tissues were fixed in 10% formalin, embedded in paraffin, and sectioned to 5-μm slides. The slides were deparaffinized and stained by using a H&E Staining Kit (Beyotime) according to the manufacturer’s protocol. For immunohistochemical staining, mouse eye tissues were first subjected to deparaffinization and antigen retrieval and then blocked with 5% BSA at 37 °C for 1 h and subsequently incubated with primary antibodies against IL-6, IFN-γ, TNF-α, and IL-1β (Proteintech Group) at 4 °C overnight. Negative controls were incubated with 0.01 M PBS. Samples were incubated with the appropriate horseradish peroxidase-conjugated secondary antibodies for 1 h at 37 °C. The SABC-HRP Kit (Beyotime) was used to visualize the sections and counterstaining with hematoxylin. The samples were observed using a Nikon fluorescence microscope (ECLIPSE Ni-U). The mean immunohistochemical staining intensity was calculated using Image Pro Plus V.6.0.

### RNA extraction and qPCR

RNA extraction was performed with Total RNA Extraction Kit (Solarbio) according to the manufacturer’s recommendations. The complementary DNA was reverse transcribed using the HiScript II Q RT SuperMix for qPCR (+gDNA wiper) (Vazyme), and qPCR was performed with SYBR Green PCR Master Mix (Vazyme) according to the manufacturer’s protocol. The mRNA levels were normalized to GAPDH expression. Primary gene-specific primer sequences are shown in Table S3.

### Western blot analysis

Corneal tissues of four eyes were collected and pooled as one sample, and three samples were used in each group. Tissue samples were lysed and the protein samples were extracted in RIPA buffer (high) (Solarbio) containing protease inhibitors. Sample protein concentrations were determined by a BCA Protein Assay Kit (Beyotime). Proteins were separated by SDS-PAGE and transferred to polyvinylidene difluoride membrane, which was incubated overnight with the appropriate primary antibody. Antibodies specific for MMP-2, MMP-9, SOD-1, and COX-2 were used: anti-MMP-2 (1:1000, Proteintech, 10373-2-AP), anti-MMP-9 (1:1000, Proteintech, 10375-2-AP), anti-SOD-1 (1:1000, Proteintech, 10269-1-AP), anti-COX-2 (1:1000, Proteintech, 12375-1-AP), and β-actin antibody (1: 10,000, Proteintech, 20536-1-AP). The secondary antibody used was HRP-conjugated goat anti-rabbit IgG (1:1000, Proteintech, SA00001-2). The bands were visualized by enzyme-linked chemiluminescence using the ECL kit (Vazyme), and the image intensity was calculated with ImageJ Software.

### Determination of cellular ROS production

Cellular ROS production was measured using a Reactive Oxygen Species Assay Kit (Beyotime) according to manufacturer's protocol. HCECs were washed twice with PBS and then incubated with 25 μM DCFDA in 1×PBS at 37 °C incubator for 20 min. After washing twice with PBS, the cells were imaged using a Nikon fluorescence microscope (ECLIPSE TI-S).

### Measurement of superoxide generation

DHE was used for the detection of ROS. Frozen sections of the corneal epithelial tissue (10 μm) were placed on glass slides and incubated with DHE (10 mmol/L, Beyotime) at 37 °C for 30 min in the dark. After rinsing in PBS three times, the sections were observed using a Nikon fluorescence microscope (ECLIPSE Ni-U).

### Statistical analysis

All statistical analyses were performed using GraphPad Prism 6, and all data were expressed as mean ± SD. The statistical significance of differences between two treatments and/or conditions was analyzed using the two-tailed unpaired Student's *t* test. When comparing more than three groups, one-way ANOVA was used. Differences were considered significant at *p* < 0.05.

## Data availability

All the data produced for this work are contained within the article.

## Supporting information

This article contains supporting information (2, 35).

## Conflict of interest

The authors declare that they have no conflicts of interest with the contents of this article.

## Supporting information

Fig. S1 Cell viability in different concentrations of glucose determined by the MTT assays. Cell viability in different concentrations of glucose was measured using the MTT assay. (∗represents statistic differences between Control and Glucose, ∗∗∗*p* < 0.001).

Fig. S2 Blood glucose levels, body weight, and tear production in STZ-induced diabetic mice. Blood glucose (*A*) and body weight (*B*) were measured at 2, 4, 6, 8 and 10 weeks after the last STZ injection. (*C*) Tear volume was measured by the Schirmer test in the Control and Diabetes groups at 10 weeks (Control group, n =12; Diabetes group, n =40; ∗*p* < 0.05, ∗∗*p* < 0.01, ∗∗∗*p* < 0.001).

Table S3 The primer sequences of the target genes.
