# Supplementary figures and images for: rhFGF-21 accelerates corneal epithelial wound healing through the attenuation of oxidative stress and inflammatory mediators in diabetic mice
Source: J Biol Chem. 2023 Aug 4;299(9):105127. doi: 10.1016/j.jbc.2023.105127 (PMC10481360; doi:10.1016/j.jbc.2023.105127)

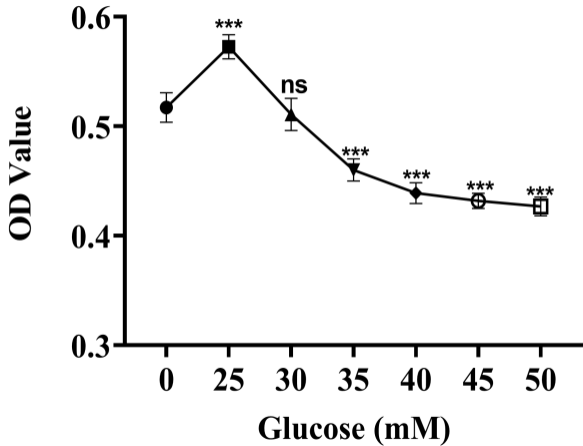

Supplement: Fig. S1 — Cell viability in different concentrations of glucose determined by the MTT assays. Cell viability in different concentrations of glucose was measured using the MTT assay. (∗represents statistic differences between Control and Glucose, ∗∗∗p < 0.001). [file mmc1.pdf]

**A**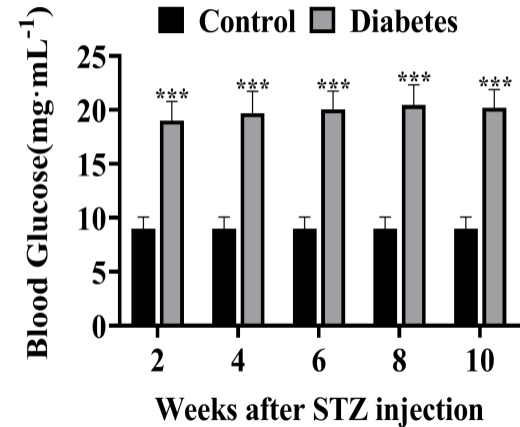**B**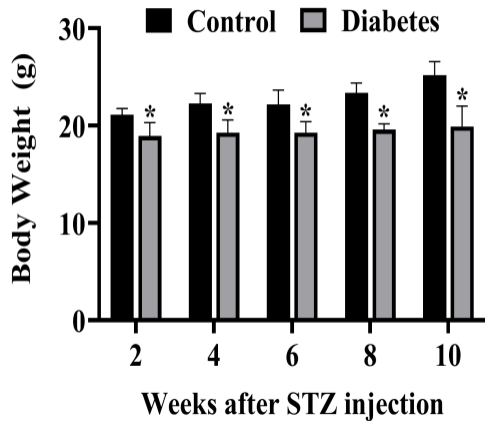**C**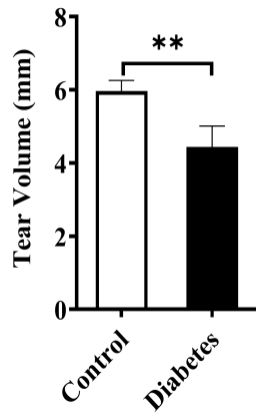

Supplement: Fig. S2 — Blood glucose levels, body weight, and tear production in STZ-induced diabetic mice. Blood glucose (A) and body weight (B) were measured at 2, 4, 6, 8 and 10 weeks after the last STZ injection. (C) Tear volume was measured by the Schirmer test in the Control and Diabetes groups at 10 weeks (Control group, n =12; Diabetes group, n =40; ∗p < 0.05, ∗∗p < 0.01, ∗∗∗p < 0.001). [file mmc2.pdf]
