# Supplementary material for: rhFGF-21 accelerates corneal epithelial wound healing through the attenuation of oxidative stress and inflammatory mediators in diabetic mice
Source: J Biol Chem. 2023 Aug 4;299(9):105127. doi: 10.1016/j.jbc.2023.105127 (PMC10481360; doi:10.1016/j.jbc.2023.105127)
Supplement: Table S3 — The primer sequences of the target genes. [file mmc3.pdf]

Supplementary Table S3

Gene	Forward (5'–3')	Reverse (5'–3')
mouse TNF-α	ACACCTGTGGCTTCATTGCAG	TAGCAGAGAGCAATAGTGTGTC
mouse MCP1	CCAGCACCAGCACCAGCCAA	TGCTCCAGCCGGCAACTGTG
mouse IFNγ	CGGCACAGTCATTGAAAGCCTA	GTTGCTGATGGCCTGATTGTC
mouse IL-10	GACCAGCTGGACAACATACTGCTAA	GATAAGGCTTGGCAACCCAAGTAA
mouse IL-1β	ATGCCACCTTTTGACAGTGATG	AGCTTCTCCACAGCCACAAT
mouse IL-6	CACAGAAGGAGTGGCTAA	GCATAACGCACTAGGTTT
human TNF-α	CCCTCACACTCAGATCATCTTCT	GCTACGACGTGGGCTACAG
human MCP1	TTAAAAACCTGGATCGGAACCAA	GCATTAGCTTCAGATTTACGGGT
human IFNγ	ATGAACGCTACACACTGCATC	CCATCCTTTTGCCAGTTCCTC
human IL-10	GCTCTTACTGACTGGCATGAG	CGCAGCTCTAGGAGCATGTG
human IL1β	AATCTGTACCTGTCCTGCGTGTT	TGGGTAATTTTTGGGATCTACACT CT
human IL-6	AAATTCGGTACATCCTCGAC	CAGGAACTGGATCAGGACTT
